# Assessment of Vitamin B12 Efficacy on Cognitive Memory Function and Depressive Symptoms: A Systematic Review and Meta-Analysis

**DOI:** 10.7759/cureus.73350

**Published:** 2024-11-09

**Authors:** Hayat Alzahrani

**Affiliations:** 1 Food Science and Nutrition, King Saud University, Riyadh , SAU

**Keywords:** cognitive memory, depression, meta-analysis, vitamin b 12, vitamin b supplementation

## Abstract

Vitamin B12 is significant for DNA synthesis, red blood cell formation, and nervous system function. Inadequate vitamin B12 levels may result in a higher risk of depression, necessitating the need for supplementation to improve mood and cognitive function. However, the use of vitamin B12 supplementation varies across studies. This review aims to evaluate the effect of vitamin B12 supplementation on cognitive memory function and depressive symptoms among participants who may have mild cognitive impairment (MCI). This review adhered to the Cochrane Handbook and PRISMA guidelines. A comprehensive search was conducted across PubMed, CENTRAL, Medline, and Ovid, with no constraints on geography or demographics, up to August 31, 2024. Only randomized controlled trials (RCTs) were included. Data on study characteristics, treatment details, and outcomes were extracted. The risk of bias was assessed using the revised Cochrane ROB2 tool. A meta-analysis of the effect of vitamin B12 on cognitive memory and depression was conducted using Jamovi software (The jamovi project (2024). jamovi (Version 2.5) [Computer Software]. Retrieved from https://www.jamovi.org). Statistical significance was set at p < 0.05. The results yielded 483 records that were screened based on Preferred Reporting Items for Systematic Reviews and Meta-Analyses (PRISMA) criteria. Excluding duplicates and irrelevant studies, the meta-analysis finally included nine RCTs. For cognitive memory function, eight of the nine included studies were analyzed, showing an average standardized mean difference of -0.03 (95% confidence interval (CI): -0.07 to 0.01), indicating no significant effect of vitamin B12 supplementation (p = 0.1801) and no significant heterogeneity. Regarding depressive symptoms, three of the nine included studies were analyzed, yielding an average standardized mean difference of -0.01 (95% CI: -0.0773 to 0.0525), also showing no significant effect (p = 0.708). These results suggest that vitamin B12 complex supplementation has an insignificant effect on cognitive function and depressive symptoms in the general population. However, further research is needed to explore the conditions under which B12 is most effective, providing clearer guidelines for its use in clinical practice.

## Introduction and background

Vitamin B12 is a crucial water-soluble vitamin that is vital in major physiological processes. These include DNA synthesis, red blood cell formation, and nervous system function. It is important for brain health, as it supports myelin formation and neurotransmitter synthesis, both essential for cognitive and emotional well-being. The vitamin B complex, including B6, B9, and B12, plays essential roles in metabolism, DNA synthesis, and nervous system function. Vitamin B6 (pyridoxine) supports amino acid metabolism, neurotransmitter production, and immune function, with sources including poultry, fish, bananas, and legumes. Vitamin B9 (folate) is crucial for DNA repair and fetal development. Vitamin B12 (cobalamin), primarily present in animal products, is vital for red blood cell formation, neurological health, and DNA synthesis. Deficiencies in B12 or folate can lead to megaloblastic anemia and neurological issues, and their metabolic pathways are closely linked [1]. Deficiency in vitamin B12 has been associated with significant neurological and psychiatric consequences, including cognitive decline, memory loss, and mood disturbances [2].

The association between vitamin B12 deficiency and cognitive impairment has been discussed in the literature. Research shows that lower vitamin B12 levels are correlated with impaired cognitive performance and reduced hippocampal volume, specifically in individuals with mild cognitive impairment (MCI) [3]. Moreover, it was found that even in cognitively healthy older adults, adequate vitamin B12 levels are positively associated with cognitive performance [4]. This suggests that maintaining optimal vitamin B12 levels may be beneficial for preserving cognitive function as individuals age. 

In addition to its impact on cognitive health, vitamin B12 is also crucial for mood regulation. Deficiency in vitamin B12 could result in an increased risk of depression among older adults [2]. For instance, Sangle et al. demonstrated that vitamin B12 supplementation can prevent the onset of depression and improve the prognosis for individuals with depressive symptoms [5]. In addition, Petridou et al. conducted a systematic review and meta-analysis revealing a significant association between low vitamin B12 levels and depression in older adults, focusing on the importance of maintaining adequate levels to support mood regulation and reduce depressive symptoms [6].

Despite the association between vitamin B12 deficiency and both cognitive and mood-related issues, the effectiveness of vitamin B12 supplementation varies across studies. While some research indicates significant improvements in cognitive function and mood with supplementation, especially in deficient populations [3,4], other studies report minimal or no effect in broader groups [1]. Considering the mixed results, a systematic review is necessary to consolidate existing evidence, assess study quality, and offer a clearer perspective on the impact of vitamin B12 on cognitive memory function and depressive symptoms. Therefore, we conducted this systematic review and meta-analysis to thoroughly evaluate the effects of vitamin B12 supplementation on cognitive memory function and depressive symptoms among patients.

## Review

Methods

The study followed the guidelines outlined in version 6 of the Cochrane Handbook for Systematic Reviews of Interventions, and the results were reported in accordance with the Preferred Reporting Items for Systematic Reviews and Meta-Analyses (PRISMA) standards [7].

Eligibility Criteria

Inclusion criteria: Double-blind randomized controlled trials (RCTs); participants either with or without mild cognitive impairment (MCI) aged above 55 years; studies investigating the therapeutic impact of a vitamin B complex on cognitive memory performance and depression; and studies published in English.

Exclusion criteria: Patients with neuropathy or advanced neurological conditions or patients with chronic medical conditions; studies that had qualitative data only; letters, reviews, editorials, observational, retrospective studies, and abstracts.

Search Strategy

We utilized the search using four databases: PubMed, CENTRAL, Medline, and Ovid to identify relevant studies published up to August 31, 2024, using subject words combined with free-text terms. The following search terms were used: “B12 vitamin,” “cyanocobalamin,” “methylcobalamin,” “cognitive function,” and “cognitive decline.” Aiming for thorough exploration, the examination of all likely applicable studies had no geographical, racial, or age constraints. Additionally, a detailed review of references listed in the retrieved articles and remarks was conducted to ensure comprehensive coverage and minimize any oversights.

Selection of Studies

One independent researcher carried out the processes of conducting online searches, screening titles and abstracts, and reviewing the full text of relevant articles.

Data Extraction

Data extracted from the included studies comprised the following: author, publication year, target population, sample size, participant age, doses of the vitamin B complex along with their frequency and route of administration, treatment duration, follow-up period, and reported outcomes.

Measured Outcomes

The effect of vitamin B complex on cognitive memory and depression among included participants.

Assessment of the Quality of the Included Studies

The quality assessment was evaluated using the revised version of risk of bias (ROB) using the ROB2 tool designed for randomized clinical trials (RCTs). The ROB2 tool consists of five domains: randomization, deviations from the assigned treatment, missing outcome data, outcome measurement, and selective reporting of outcomes and results. Additionally, the overall ROB is determined by identifying the highest level of ROB among the five domains [8]. The Robvis tool was utilized to visualize the ROB figures [9].

Data Synthesis

All data analyses were performed using Jamovi software (The jamovi project (2024). jamovi (Version 2.5) [Computer Software]. Retrieved from https://www.jamovi.org) [10]. Different clinical tests were used to measure the outcomes of “cognitive memory" and "depression." Consequently, we conducted a meta-analysis of the results of each test. The findings from the meta-analyses for each outcome were presented in forest plots, illustrating the estimated effects of the vitamin B complex and the overall effect of B12 supplementation. We used the standardized mean difference to estimate the outcomes. Heterogeneity was assessed using the restricted maximum-likelihood estimator, and both the Q-test for heterogeneity and the I² statistic were reported. A p-value threshold of 0.05 was established for determining statistical significance.

Results

Characteristics of the Included Studies

**Figure 1:**
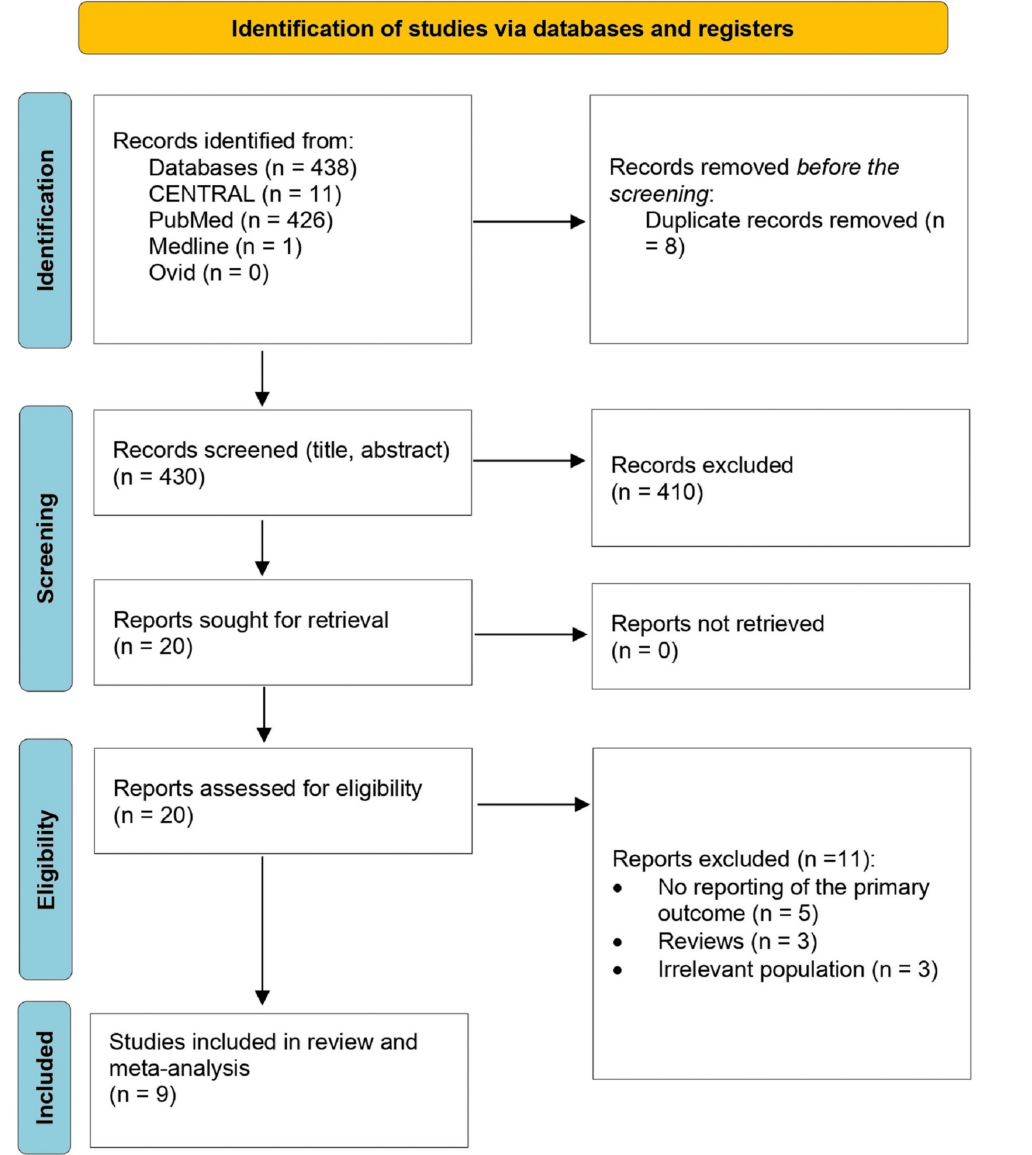
PRISMA flowchart of the included studies PRISMA: Preferred Reporting Items for Systematic Reviews and Meta-Analyses

The process of screening and selecting the included studies was based on PRISMA criteria (Figure 1). The retrieving process yielded 438 studies. Eight studies were excluded as duplicates. The remaining 430 studies were screened for their titles and abstracts. Out of them, 410 studies were excluded based on their titles and abstracts. These exclusions were made because the studies did not meet the pre-specified inclusion criteria and were not relevant to the research topic. The remaining 20 records were reviewed for full text. During the exclusion process based on the eligibility criteria, five studies were excluded due to incomplete reporting of primary outcomes, three were reviews, and three had inappropriate populations. Finally, nine articles were included for the review and meta-analysis [11-19].

Characteristics of the Included Studies

The nine included studies focused on the effects of vitamin B12 and B9 (folate) supplementation on cognitive outcomes in populations with and without mild cognitive impairment (MCI). The sample sizes vary significantly, ranging from 179 to 2,919 participants. Participants' mean ages were generally in the elderly range, from 60 to 82.5 years. Most studies reported baseline vitamin B12 serum levels. The vitamin B12 doses administered daily were between 500 and 1,000 mcg, combined with 400 mcg of vitamin B9 and, in some cases, vitamin B6. Treatment durations extended from 24 to 104 weeks. Table 1 shows the characteristics of the included studies.

**Table 1:** Characteristics of the included studies NA: not available; NR: not reported; MCI: mild cognitive impairment *median [IQR]; **: extensive cognitive tests subsample

Author, year	Sample size (N)	Female n (%)	Mean age (SD), years	Population condition	Vitamin B12 serum level (SD) for intervention (I), pmol/L	Vitamin B12 serum level (SD) for for placebo (P), pmol/L	Vitamin B12 daily defined dose administration, mcg	Vitamin B9 administration, mcg	Vitamin B6 administration, mg	Intake frequency, route of administration	Treatment duration, follow-up duration, weeks	Outcome
Kwok, 2020 [11]	279	113 (40.5)	77.5 (NA)	MCI	NR	NR	500 (500)	400	NA	Daily, oral	52, 104	Cognitive, depression
de Koning, 2016 [12]	2919	1459 (50.0)	74.1 (n.a.)	No MCI	267.0 (213.0–341.0) *	266.0 (204.0–343.0)*	500 (500)	400	NA	Daily, oral	104,104	Depression
van der Zwaluw, 2014 [13]	2919	1459 (50.0)	74.1 (6.5)	No MCI	I1: 267.0 (231.0–341.0)*; I2**: 257.0 (200.0–326.0)*	P1: 266.0 (204.0–343.0)*; P2**: 263.0 (200.0–345.0)*	500 (500)	400	NA	Daily, oral	104,104	Cognitive
Walker, 2012 [14]	900	542 (60.2)	66.0 (4.3)	No MCI	305.32 (151.05)	285.27 (105.77)	100 (100)	400	NA	Daily, oral	104, 104	Cognitive
Ford, 2010 [15]	299	0 (0.0)	79.0 (2.8)	No MCI	256.12 (121.86)	253.02 (115.35)	400 (400)	2000	25	Daily, oral	104, 104	Cognitive
Walker, 2010 [16]	900	542 (60.2)	66.0 (4.3)	No MCI	305.32 (151.05)	285.27 (105.77)	100 (100)	400	NA	Daily, oral	104, 104	Depression
van Uffelen, 2008 [17]	179	67 (37.4)	75.17 (n.a.)	MCI	NR	NR	400 (500)	5000	50	Daily, oral	52,52	Cognitive
Eussen, 2006 [18]	195	100 (51.3)	82.5 (6.0)	No MCI to MCI	199.0 (50.0)	188.0 (56.0)	1000 (1000)	400	NA	Daily, oral	24,24	Cognitive
McMahon, 2006 [19]	276	112 (44.3)	73.5 (5.8)	No MCI	380.0 (136.0)	385.0 (138.0)	500 (500)	1000	10	Daily, oral	104,104	Cognitive

Risk of Bias (ROB) Assessment

**Figure 2:**
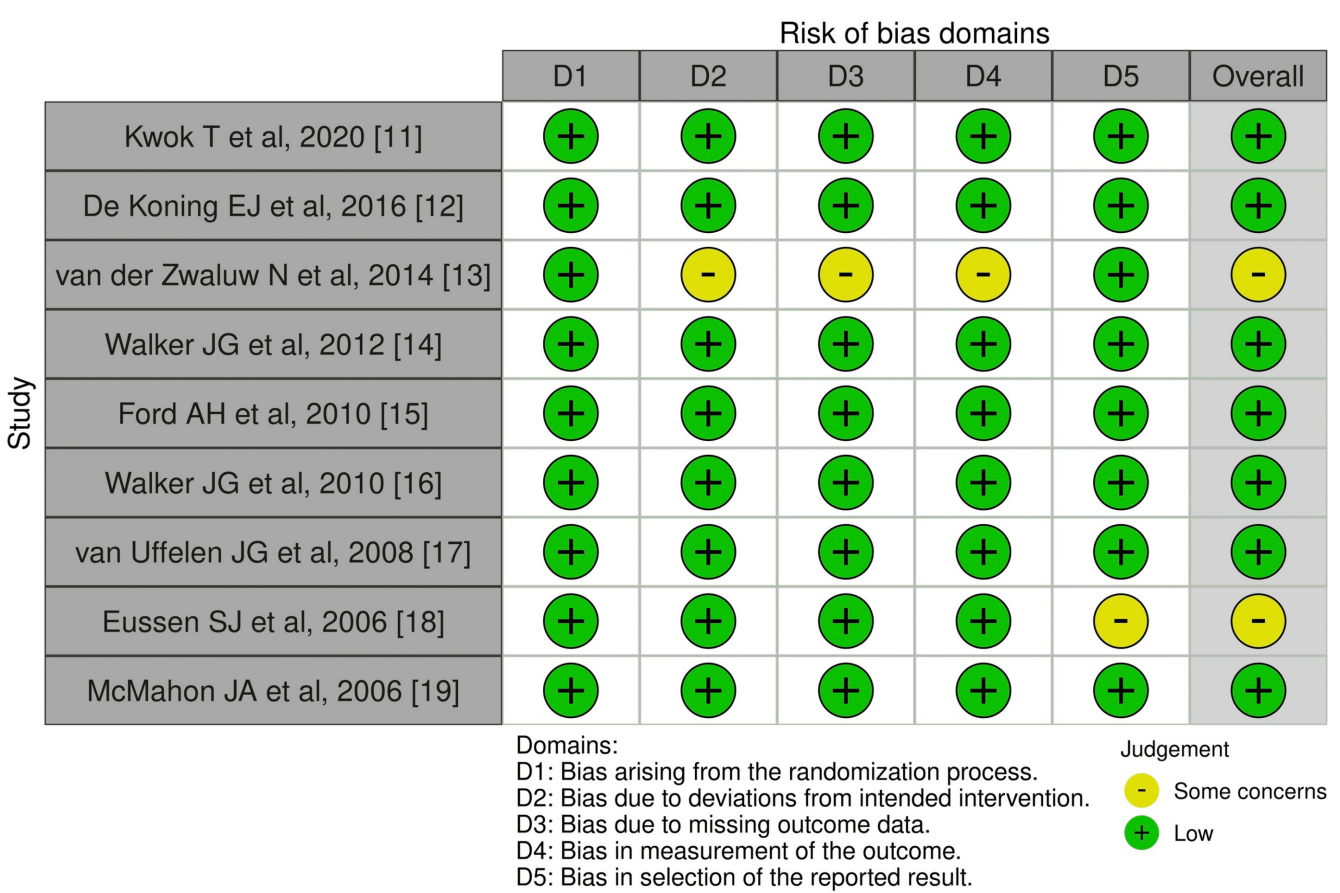
Risk of bias (ROB) assessment [11,12,13,14,15,16,17,18,19]

**Figure 3:**
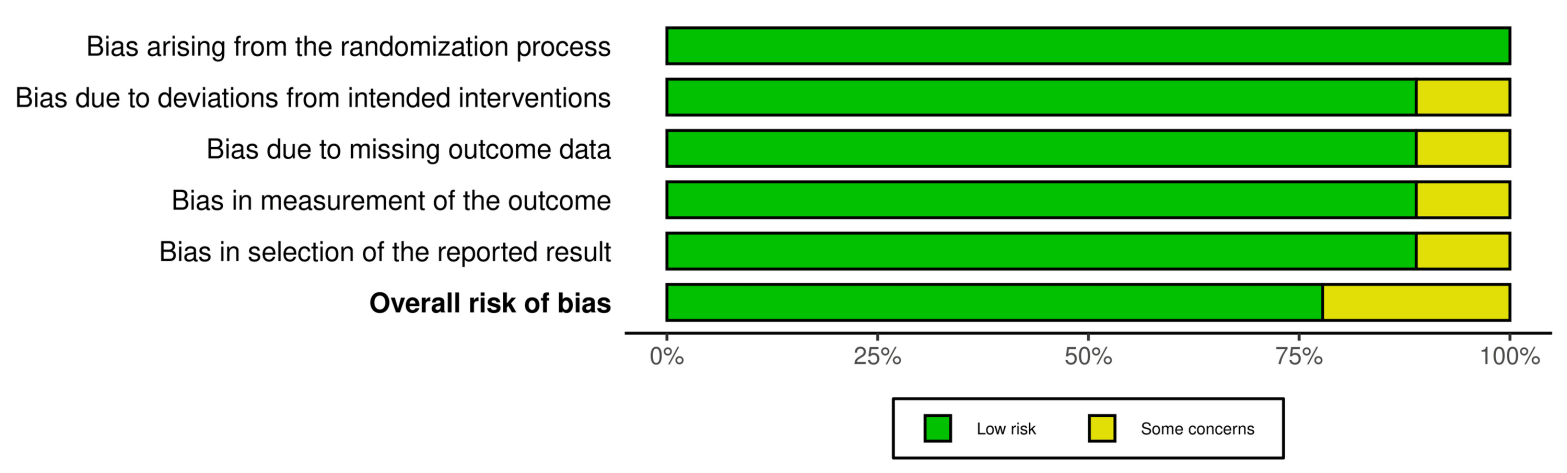
Risk of bias (ROB) summary

The risk of bias assessment indicated a low risk across all domains, except for two studies that raised concerns regarding deviations from intended interventions, missing outcome data, outcome measurement, and selective reporting (Figures 2, 3) [13,18].

Meta-analysis

**Figure 4:**
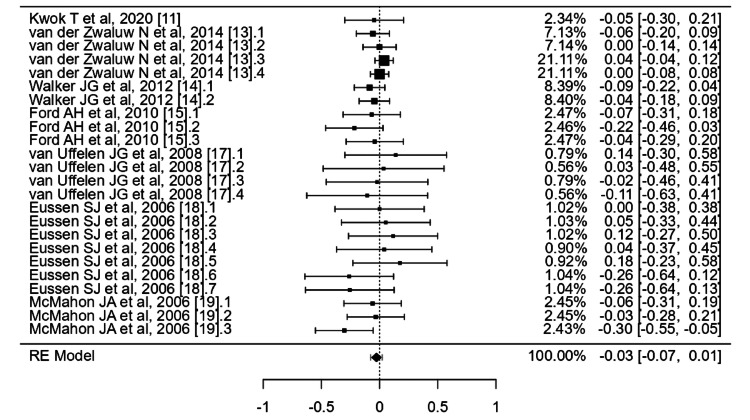
Forest plot for effects of vit B12 complex on cognitive memory function AVLT: auditory verbal learning test; CPAL: continuous paired associates learning; CVLT: California verbal learning test; DCT: digit cancellation test; ISLT: international shopping list test; RAVLT: Rey auditory verbal learning test; TICS-M: telephone interview for cognitive status modified The outcome measurement method for each trial was as follows: Kwok T et al., 2020: ISLT, CPAL; van der Zwaluw N et al., 2014: (1) Digit Span Backward; (2) Digit Span Forward; (3) RAVLT (Immediate Recall); (4) RAVLT (Recognition); Walker JG et al., 2012: (1) TICS-M (Delayed Recall Score); (2) TICS-M (Immediate Recall Score); Ford AH et al., 2010: (1) CVLT (A Long-Delay Free Recall); (2) CVLT (Trial 1-5); (3) DCT; van Uffelen JG et al., 2008: (1) AVLT (Trial 6) (Men); (2) AVLT (Trial 6) (Women); (3) AVLT (Trials 1-5) (Men); (4) AVLT (Trials 1-5) (Women); Eussen SJ et al., 2006: (1) 15 Word Learning Test (Delayed Recall); (2) 15 Word Learning Test (Immediate Recall); (3) 15 Word Learning Test (Recognition); (4) Complex Figure of Rey (Delayed Recall); (5) Complex Figure of Rey (Immediate Recall); (6) Digit Span Backward; (7) Digit Span Forward; McMahon JA et al., 2006: (1) RAVLT (Trial 7); (2) RAVLT (Tria ls 1-5); (3) Wechsler Paragraph Recall Test. [11,13,14,15,17,18,19]

Regarding the effect of vitamin B12 complex on cognitive memory function, seven studies were included in the analysis (Figure 4). The standardized mean differences observed ranged from -0.3026 to 0.1753, with a majority of the estimates (58%) being negative. The average standardized mean difference estimated by the random-effects model was -0.03 (95% confidence interval (CI): -0.07 to 0.01). Thus, there was no significant difference in the average outcome (p = 0.1801). The Q-test indicated no significant heterogeneity in the true outcomes (I^2^ = 20%, p = 0.8175). The 95% CI for the true outcomes ranged from -0.0761 to 0.0229.

**Figure 5:**
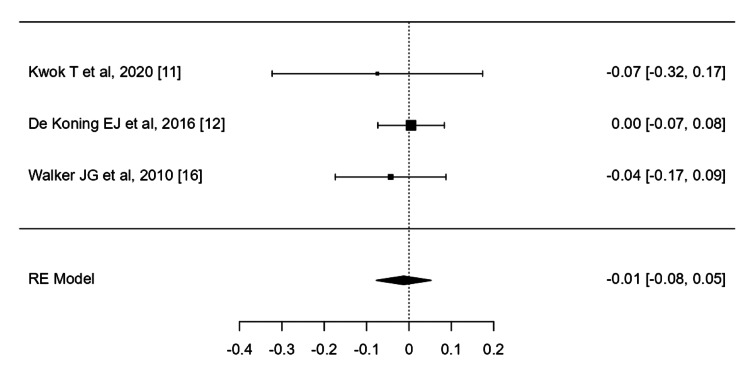
The effect of vit B12 complex on depression BDI: Beck Depression Inventory; HDRS: Hamilton depression rating scale; K10: Kessler psychological distress scale; MDI: major depression inventory; PHQ-9: patient health questionnaire 9; SF-12 MCS score: 12-item short-form health survey mental component summary The outcome measurement method for each trial was as follows: Kwok T et al., 2020: HDRS; de Koning EJ et al., 2016: SF-12 MCS score; Walker JG et al., 2010: PHQ-9 [11,12,16]

The effects of B12 complex on depressive symptoms were measured using the standardized mean difference through a random-effects model. Heterogeneity was assessed using the restricted maximum-likelihood estimator, and both the Q-test for heterogeneity and the I² statistic were reported (I^2^ = 25%). A total of three studies were included in this analysis (Figure 5). The average standardized mean difference estimated by the random-effects model was -0.01 (95% CI: -0.0773 to 0.0525), indicating that the average outcome did not significantly differ from zero (p = 0.708). The Q-test results showed no significant heterogeneity in the true outcomes (p = 0.7256).

Discussion

The meta-analysis of the effects of the vitamin B12 complex on cognitive memory function showed a range of standardized mean differences. A random-effects model indicated no significant effect of B12 on cognitive memory function. Similar analyses of depressive symptoms also showed no significant effect of B12, with no observed heterogeneity. These findings suggest that B12 supplementation may not have a substantial impact on cognitive or emotional health in general populations without specific deficiencies or underlying conditions.

Cognitive Memory Function

The meta-analysis of vitamin B12's complex effect on cognitive memory function shows small and statistically insignificant effects, with an average standardized mean difference. These findings indicate that B12 complex supplementation has a negligible influence on memory function in the general population, which is aligned with the results of Agnew-Blais et al. (2015) [20], as it found no significant association between B12 intake and reduced risk of mild cognitive impairment (MCI) or dementia among women. These results suggest that B12's influence on cognitive health may not be strong in populations without severe deficiencies or neurological issues.

However, studies targeting specific conditions are inconsistent with these findings. For instance, Akbari et al. [21] indicated that B12 administration could prevent ethanol-induced cognitive impairment by restoring brain antioxidant balance and enhancing brain-derived neurotrophic factor (BDNF) expression. Similarly, Bito et al. showed that B12 deficiency resulted in severe oxidative stress and memory retention impairment in Caenorhabditis elegans [22]. These results suggest that while B12 may not enhance cognition in the general population, it has a role in preventing cognitive decline under oxidative stress or neurotoxic cases.

This contrast suggests that the effects of the B12 complex may be context-dependent. B12 complex supplementation might not be beneficial in populations without specific risk factors, but it may offer neuroprotective benefits in individuals exposed to oxidative stress, neuroinflammation, or neurotoxic substances.

Effect of Vitamin B12 Complexes on Depression: Broader Evidence vs. Targeted Findings

There was no significant effect of B12 on depressive symptoms. These findings suggest that B12 has a negligible effect on mood in the general population.

However, when examining specific populations, studies reveal a clearer picture. Todorov et al. [23] found a significant correlation between low levels of B12 levels and symptoms of depression and anxiety among patients when compared to healthy controls. This indicates that B12 deficiency may affect mood-related conditions and that supplementation could help individuals with such diagnosed deficiencies. Additionally, Mikkelsen et al. [24] showed that the effects of B vitamins on depression played a role in reducing depressive symptoms, especially when targeting individuals with high homocysteine levels or B12 deficiencies. 

These findings suggest that B12 supplementation could be a valuable adjunct in treating depression in individuals with specific nutritional deficiencies or elevated homocysteine levels. This dependent efficacy reflects the findings for cognitive function and reinforces the argument that B12's effects are more evident in populations with vulnerabilities.

Limitations

While the findings on vitamin B12’s effects on cognitive function and depression provide useful insights, several limitations related to the co-administration of additional vitamins must be considered. All included studies supplemented vitamin B9 (folate) alongside B12, with some also including vitamin B6, introducing potential confounding effects. Folate plays an essential role in methylation and neural development, and its synergistic function with B12 makes it challenging to isolate the independent impact of B12 on cognitive outcomes. In several studies, the dosage of vitamin B9 was higher than that of B12, which may have disproportionately influenced results, as excessive folate can mask B12 deficiency and complicate the interpretation of cognitive and neurological outcomes. Moreover, variability in dosages across studies and differences in participants' baseline nutritional status further limit the generalizability of the findings. The inconsistency in cognitive and mood assessments, where studies employed diverse tools for memory and different depression scales, adds another layer of complexity, potentially contributing to discrepancies in outcomes rather than reflecting the true efficacy of B12. Addressing these limitations in future research could help clarify the individual contributions of each vitamin.

Clinical Implications

The findings emphasize the importance of personalized management using B12 complex supplementation. Although the meta-analyses suggest minimal benefit for cognitive function and depressive symptoms, individuals with B12 deficiencies, high homocysteine levels, or specific neuroinflammatory conditions may benefit from B12 complex supplementation. The findings highlight the need for assessment of patients' nutritional status. Thus, measuring B12 and homocysteine levels and identifying factors that may cause B12 malabsorption can help define patients who might respond to B12 complex supplementation.

Future research should focus on tailoring supplementation strategies to individual nutritional needs. This includes conducting large-scale studies to assess the baseline nutritional status of patients, particularly measuring B12 and homocysteine levels. Identifying factors that may contribute to B12 malabsorption, such as gastrointestinal disorders, medications, or dietary habits, will further refine the selection of patients who are most likely to benefit from B12 complex supplementation. Investigating the interplay between B12 supplementation and other nutrients, particularly folate and B6, could also provide insights into optimizing treatment protocols for cognitive health and mood disorders.

## Conclusions

In conclusion, the results suggest that vitamin B12 complex supplementation has an insignificant effect on cognitive function and depressive symptoms in the general population. While this supplementation may not be universally effective for cognitive or mental health, it may be effective among specific populations. This underscores the importance of personalized medicine in nutrition and mental health treatment, where interventions should be specified to individuals rather than to the general population. Further research should explore the conditions under which B12 is most effective, providing clearer guidelines for its use in clinical practice.
